# Anti-inflammatory activity of Yanshu spraying agent in animal models

**DOI:** 10.3892/etm.2012.761

**Published:** 2012-10-25

**Authors:** JIANQIAO ZHANG, HONGSHENG WANG, TIAN WANG, YATING CHONG, PENGFEI YU, CHENGWEN LU, YUNLI XUE, FENGHUA FU, LEIMING ZHANG

**Affiliations:** 1 Department of Pharmacology, School of Pharmacy, Yantai University, Yantai, Shandong 264005;; 2 Nanjing Luye Sike Pharmaceutical Co., Ltd, Nanjing, Jiangsu 210061, P.R. China

**Keywords:** acute pharyngitis, Yanshu spraying agent, inflammation, prostaglandin E2, cycloxygenase-2

## Abstract

Acute pharyngitis is characterized by an inflammation of the mucous membranes in the pharynx. Yanshu spraying agent was prepared according to the traditional Chinese formulation for the treatment of acute pharyngitis. The present study aimed to investigate the anti-inflammatory effect of Yanshu in xylene-induced ear edema in mice and carrageenan-induced paw edema in rats by measuring the degree of edema in the animal models. The histopathology and the levels of prostaglandin E2 (PGE2) and cycloxygenase-2 (COX-2) in the hind paws of the carrageenan-treated rats were also analyzed. The results showed that Yanshu significantly reduced ear edema in the mice and paw edema in the rats. Furthermore, treatment with Yanshu also reduced the number of inflammatory cells in tissue and decreased the production of PGE2 and COX-2. These results suggest that Yanshu possesses potent anti-inflammatory activity mediated by the inhibition of COX-2 expression which, in turn, downregulates the inflammatory mediator PGE2.

## Introduction

Acute pharyngitis, an acute inflammation of the pharyngeal mucous membrane, is often caused by upper respiratory tract infection with a virus or bacteria. Its clinical manifestations include soreness of the throat, cough, pain on swallowing and fever (1).

The most common causes of acute pharyngitis are viral infections (2). Normally, the illness is mild and self-limiting but it may worsen due to bacterial superinfection, most commonly by group A or C β-hemolytic streptococci. Serious sequelae, including rheumatic fever and endocarditis, may follow bacterial superinfection (1). Due to the predominantly viral cause, antibiotics have only a minor benefit upon the resolution of pharyngitis symptoms. Thus, in most cases the treatment of acute pharyngitis is symptomatic, focusing on the management of symptoms such as fever, pain and inflammation.

Previous treatments of acute pharyngitis with fever, pain or inflammation include non-steroidal anti-inflammatory drugs and steroids (3,4), which have potential risks and side-effects (5). Chinese traditional medicine has been used to treat pharyngitis for a number of years (6,7). The treatment principle for this disease depends upon its symptoms or cause. Anti-inflammatory, antiviral and fever relieving herbs are the basic elements of the prescription.

Yanshu spraying agent, a potential drug for pharyngitis, consists of eleven herbs, including *Radix Sophorae Tonkinensis*, *Calyx seu Fructus Physalis Francheti* and *Radix Scutellariae Baicalensis*. However, its pharmacological effects have not been demonstrated. The aim of this study was to investigate the anti-inflammatory effects of Yanshu in the treatment of xylene- and carrageenan-induced acute inflammation in animals.

## Materials and methods

### Drugs and chemicals

Yanshu was provided by Shandong Luye Pharmaceutical Co., Ltd. (Yantai, China; batch no. 20110810). It is composed of 11 plant materials: *Radix Sophorae Tonkinensis*, *Calyx seu Fructus Physalis Francheti*, *Periostracum Cryptotympanae*, *Radix Rehmanniae*, *Fructus Arctii*, *Radix Scutellariae Baicalensis*, *Radix Paeoniae Rubra*, *Semen Oroxyli*, *Semen Sterculiae Lychnophorae*, *Cortex Moutan Radicis* and *Herba Menthae Haplocalycis* in a ratio of 1.7:1.7:1.7:2.4:1.7:1.7:2:1.7:1.7:1.7:1.7 (by dry weight). All plant materials were chosen according to the Pharmacopoeia of the People’s Republic of China (2010). Yanshu (2 kg) was prepared as a mixture of all the above components and extracted twice with distilled water (24 and 20 l) at 100°C for 1.5 h. After filtering, the liquid extract was concentrated with a rotary vacuum evaporator to a concentration of 2.0 g/ml and stored at 4°C.

Diclofenac diethylamine emulsion (Votalin) was purchased from Novartis Pharmaceutical Co. (Beijing, China; batch no. X2347); prostaglandin E2 (PGE2) and cycloxygenase-2 (COX-2) ELISA assay kits were purchased from Shanghai Xitang Biological Technology Co., Ltd. (Shanghai, China; batch nos. 1206122 and 1204251); xylene was purchased from Tianda Chemical Factory (Tianjin, China); and λ-carrageenan was purchased from Sigma-Aldrich (Shanghai, China; batch no. 1408463V).

### Animals

Mice weighing 18–22 g and male Wistar rats weighing 160–200 g were purchased from the Experimental Animal Center of Shandong Luye Pharmaceutical Co., Ltd. All experimental procedures carried out in this study were performed in accordance with the guidelines for the care and use of laboratory animals of Yantai University, and were approved by the Ethics Committee. All the animals were housed in diurnal lighting conditions (12/12 h) and allowed free access to food and water.

### Xylene-induced ear edema model

Forty Swiss mice were randomly divided into control, Votalin and two Yanshu groups (40 and 80 *μ*l doses), each containing 10 animals. Both ears of each mouse were smeared with saline, 50 mg Votalin, 40 *μ*l Yanshu (Yanshu 40) or 80 *μ*l Yanshu (40 *μ*l twice at intervals of 30 min; Yanshu 80). Xylene (20 *μ*l) was dropped onto the anterior and posterior surfaces of the right ear 30 min after the final administration. The mice were sacrificed 1 h after the application of xylene. An ear disc of 9.0-mm diameter was punched out of each ear and weighed. The level of edema was determined from the weight difference between the right (treated) and the left (control) ear discs of the same animal. The inhibition level (%) was calculated according to the following equation: Inhibition (%) = (1−Et/Ec) × 100, where Et is the average edema of the treated group and Ec is the average edema of the saline-treated control group.

### Carrageenan-induced paw edema model

Forty Wistar rats were randomly divided into control, Votalin, Yanshu 200 *μ*l (100 *μ*l twice at intervals of 15 min; Yanshu 200) and Yanshu 400 *μ*l (100 *μ*l 4 times at intervals of 15 min; Yanshu 400) groups. The right-hind paw of each rat was then smeared with saline, Votalin or Yanshu. Carrageenan (0.1 ml, 1%w/v) was injected into the right-hind paw (sub-plantar) 30 min after the final administration. The paw volume was measured prior to the irritant injection and at selected intervals (0.5, 1, 1.5, 2, 2.5, 3, 4 and 5 h) thereafter with a hydro-plethysmometer (Shandong Academy of Medical Sciences, Shandong, China). Results were expressed according to the increase in paw volume (ml) calculated by subtracting the basal volume.

### Hematoxylin and eosin (H&E) staining

Three hours after carrageenan injection, the rats were anesthetized with chloral hydrate and tissues of the right-hind paw were excised and immersed into 4% formaldehyde for 24 h. The tissues were then embedded in paraffin, sectioned (5 *μ*m) using a microtome and stained with H&E. The pathological features were determined by analysis under a microscope (magnification, x400; Olympus BX41, Tokyo, Japan).

### PGE2 and COX-2 assay

Three hours after carrageenan injection, the rats were anesthetized with chloral hydrate and tissues of the right-hind paw were separated on ice and homogenized with ice-cold saline to form a 10% (w/v) homogenate. The levels of PGE2 and COX-2 in the paw were determined using an ELISA kit.

### Statistical analysis

The data are expressed as mean ± SD. Data was analyzed using one-way ANOVA with Bonferroni post hoc test for multiple t-tests. P<0.05 was considered to indicate a statistically significant result.

## Results

### Effects of Yanshu on xylene-induced ear edema in mice

**Figure 1:**
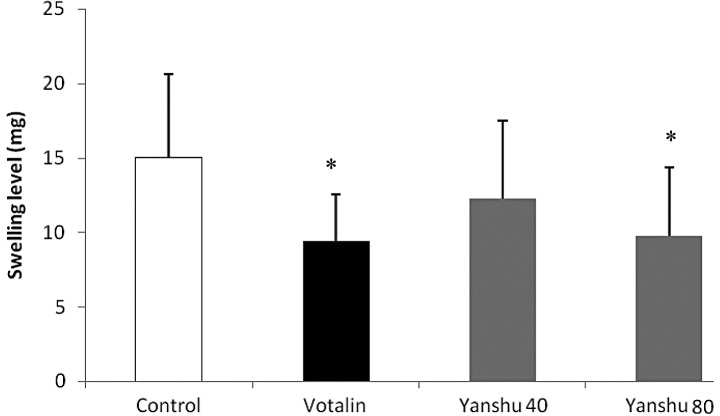
Effect of Yanshu on xylene-induced ear edema in mice. Results are expressed as mean ± SD (n=10). ^*^P<0.05 compared with the control group.

The treatment of the mice with Yanshu 80 but not Yanshu 40 suppressed xylene-induced ear edema significantly, reducing swelling by 34.7% compared with the control group. Votalin administration decreased swelling by 37.3% 1 h after the induction of ear edema (Fig. 1).

### Effects of Yanshu on carrageenan-induced paw edema in rats

**Figure 2:**
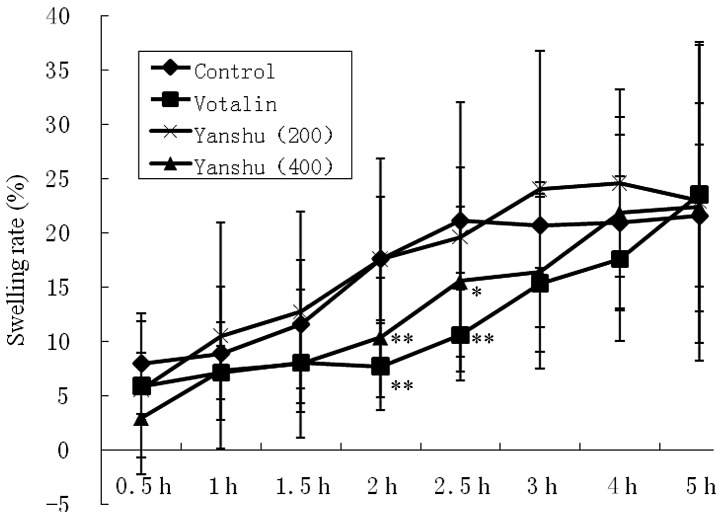
Effect of Yanshu on carrageenan-induced paw edema in rats. Results are expressed as mean ± SD (n=10). ^*^P<0.05, ^**^P<0.01 compared with the control group.

The treatment of rats with Yanshu 400 but not Yanshu 200 significantly inhibited the development of paw edema at 2 h (P<0.01) and 2.5 h (P<0.05) after carrageenan injection. Votalin also significantly decreased the carrageenan-induced paw edema at 2 h (P<0.01) and 2.5 h (P<0.01) after carrageenan injection (Fig. 2).

### Effects of Yanshu on histological pathology of the paws in rats

**Figure 3:**
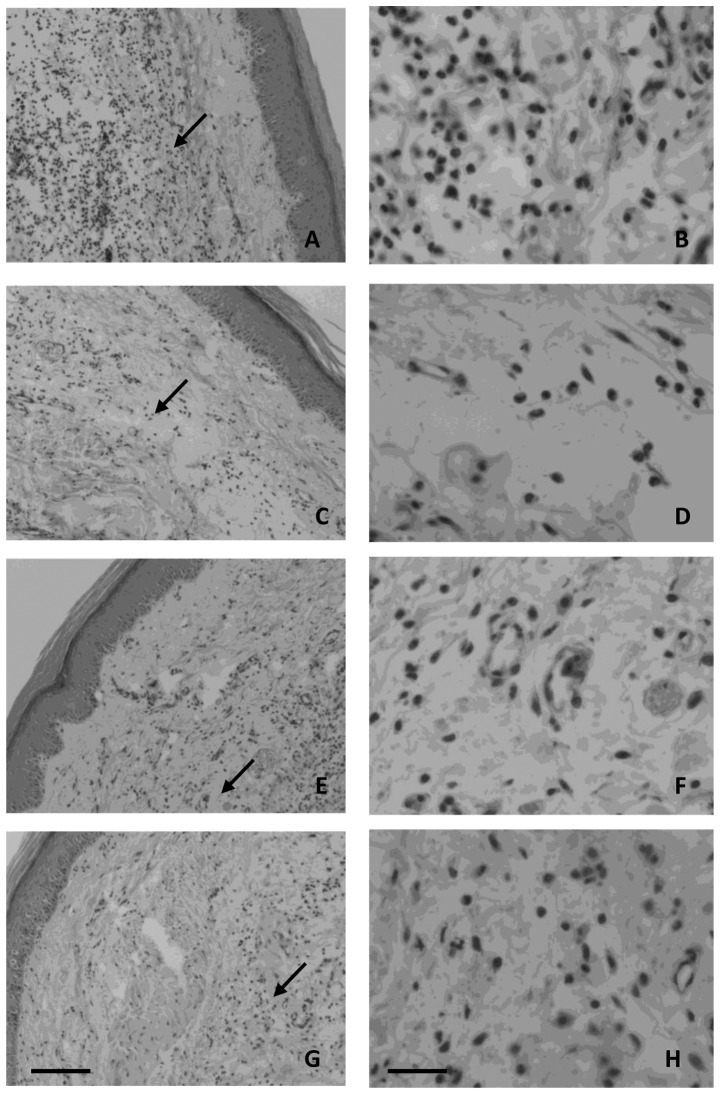
Histopathology of the swollen paw induced by carrageenan in rats. Light photomicrographs of the hematoxylin and eosin stained paw tissue in the (A and B) control, (C and D) Votalin, (E and F) Yanshu 200 and (G and H) Yanshu 400 groups. Arrows indicate the area examined with a high power microscope. Scale bar: A, C, E and G, 200 *μ*m; B, D, F and H, 40 *μ*m.

A large number of inflammatory cells were observed in the paw tissue 3 h after carrageenan injection and the number of neutrophils increased significantly (Fig. 3A and B). Compared with the control group, Votalin and Yanshu treatment, particularly at the higher dose, reduced the number of inflammatory cells significantly (Fig. 3C, D, G and H).

### Effects of Yanshu on the PGE2 levels of the paws in rats

**Figure 4:**
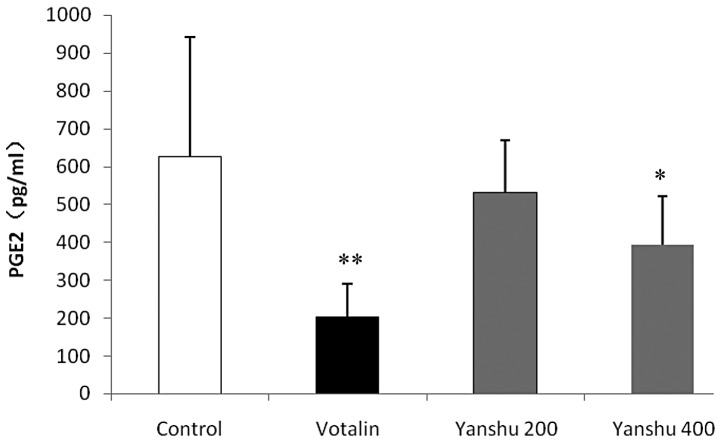
Effect of Yanshu on PGE2 production in the paw tissue of rats induced by carrageenan. Results are expressed as mean ± SD (n=10). ^*^P<0.05, ^**^P<0.01 compared with the control group. PGE2, prostaglandin E2.

The PGE2 level increased significantly in the paw tissue 3 h after carrageenan injection. Votalin and Yanshu 400 decreased the PGE2 level significantly 3 h after carrageenan injection (P<0.01 and P<0.05, respectively; Fig. 4).

### Effects of Yanshu on COX-2 level of the paws in rats

**Figure 5:**
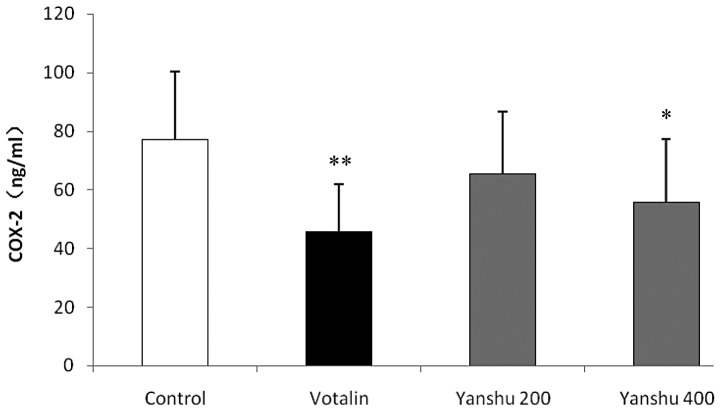
Effect of Yanshu on the COX-2 production in paw tissues of rats induced by carrageenan. Results are expressed as mean ± SD (n=10). ^*^P<0.05, ^**^P<0.01 compared with the control group. COX-2, cyclooxygenase-2.

The COX-2 level increased significantly in the paw tissue 3 h after carrageenan injection. Votalin and Yanshu 400 decreased the COX-2 level significantly 3 h after carrageenan injection (P<0.01 and P<0.05, respectively; Fig. 5).

## Discussion

In the present study, we examined the *in vivo* anti-inflammatory effect of Yanshu using two common animal models of inflammation, xylene-induced ear edema in mice and carrageenan-induced paw edema in rats. In addition, the histopathology and inflammatory mediators, including PGE2 and COX-2, were determined to illuminate the underlying anti-inflammatory mechanisms of action.

Xylene-induced ear edema in mice is a simple animal model for evaluating potential anti-inflammatory agents (8,9). In our study, xylene-induced ear edema led to fluid accumulation and edema, characteristic of the acute inflammatory response. Treatment of the mice with Votalin and a high dose of Yanshu suppressed xylene-induced ear edema, reducing swelling by 37.3 and 34.7%, respectively. The data indicate that Yanshu possesses inhibitory effects against acute inflammation.

Carrageenan-induced paw edema is another model which is widely employed for screening the effects of anti-inflammatory drugs (10,11). The acute inflammatory response induced by carrageenan injection involves two phases (12). The early phase occurs during the first hour of exposure and is associated with the release of histamine, serotonin, bradykinin and, to a lesser extent, prostaglandins (PGs). The delayed phase after one hour is attributed to polymorphonuclear (PMN) leucocyte infiltration and the continuation of PG generation. In the present study, a large number of inflammatory cells were observed in the paw tissue 3 h after carrageenan injection and the number of neutrophils increased significantly. Compared with the control group, Votalin and Yanshu treatment, particularly the higher dose, reduced the number of inflammatory cells significantly. These data support the results from the ear edema assay and verify the anti-inflammatory effect of Yanshu against acute inflammation.

It is well established that PGs, by virtue of their activity as modulators of inflammatory responses, have a major role in the inflammatory process. COX is the key enzyme that synthesizes PGs and thromboxane from arachidonic acid. Inappropriate COX-2 expression is known to be involved in the development of inflammatory pathogenesis seen in diseases of the gastrointestinal tract and central nervous system, ischemia and lung inflammation and fibrosis (13,14). In the present study, treatment with Yanshu significantly inhibited the paw edema 2–2.5 h after carrageenan injection. In addition, Yanshu also decreased the production of PGE2 and COX-2 in the hind paws of carrageenan-treated rats; this indicated that Yanshu exerted an anti-inflammatory effect by inhibiting the PGE2/COX-2 inflammatory pathway.

In conclusion, the present study demonstrated that Yanshu possesses anti-inflammatory activities that may be mediated through the regulation of the PGE2/COX-2 inflammatory pathway. These findings indicate that Yanshu may be of benefit for the treatment of acute pharyngitis in the clinic.
